# Supplementary material for: In Vitro Susceptibility to Imipenem/Relebactam and Comparators in a Multicentre Collection of Mycobacterium abscessus Complex Isolates
Source: Antibiotics (Basel). 2025 Jul 5;14(7):682. doi: 10.3390/antibiotics14070682 (PMC12291787; doi:10.3390/antibiotics14070682)
Supplement: Supplementary file 1 [file antibiotics-14-00682-s001.zip › Supplementary material Table S1.pdf]

Table S1. Antimicrobial agents used in the study and MIC breakpoints.

	MIC (ug/mL)		
	**S**	**I**	**R**
**Amikacin**	≤16	32	≥64
**Clarithromycin**	≤2	4	≥8
**Moxifloxacin**	≤1	2	≥4
**Linezolid**	≤8	16	≥32
**Clofazimine**	≤1	2	≥4
**Tigecycline**	≤4		>4
**Imipenem**	≤4	8-16	≥32
